# Examining Social Determinants of Health, Stigma, and COVID-19 Disparities

**DOI:** 10.3390/healthcare8020168

**Published:** 2020-06-12

**Authors:** Jocelyn Turner-Musa, Oluwatoyin Ajayi, Layschel Kemp

**Affiliations:** 1 Department of Psychology, Morgan State University, Baltimore, MD 21251, USA; lakem1@morgan.edu; 2 ASCEND Center for Biomedical Research, Morgan State University, Baltimore, MD 21251, USA; oluwatoyin.ajayi@morgan.edu; 3 School of Community Health and Policy, Morgan State University, Baltimore, MD 21251, USA

**Keywords:** health disparities, COVID-19, stigma, social determinants of health, race/ethnicity

## Abstract

There is growing attention to disparities in the incidence, prevalence, and mortality associated with COVID-19 (Coronavirus disease 2019) in racial/ethnic communities. The conditions leading to these disparities may be a function of social determinants of health and stigma linked to the disease. It is important to examine how these factors may be implicated in COVID-19 onset, treatment, and outcomes. A brief overview of these issues allows for a cursory examination of the role of social determinants of health and stigma in COVID-19. Consideration is given to how understanding COVID-19 in the context of social determinants and stigma may be included in interventions to mitigate its transmission within vulnerable populations.

## 1. Introduction

The 2019 novel coronavirus (2019-nCoV), also known as SARS-Cov-2 (Severe Acute Respiratory Syndrome Coronavirus 2), is one of seven types of coronaviruses that infects humans, typically leading to serious upper respiratory problems causing COVID-19 disease. Transmission of the virus occurs through the air via coughing and sneezing, close personal contact with someone infected with the virus, and touching an object or surface contaminated with the virus [1]. The virus originated in Wuhan, China, where, in December 2019, a cluster of 27 cases of pneumonia of unknown etiology was observed [2,3]. The pneumonia cases were linked to a new strain of the coronavirus, which research suggests was found in infected animals and initially transmitted to humans in an open-air seafood/animal market in Wuhan, China [4]. Since December 2019, the illness has spread to 213 countries and territories and was declared a “Public Health Emergency of International Concern” in January 2020 and a pandemic by the World Health Organization in March 2020 [5]. As of 4 June 2020, there are 6,416,828 cases of COVID-19 worldwide and have been 382,867 deaths (Table 1) [6]. The countries with the highest prevalence and mortality rates are the United States with 1,823,220 cases and 106,051 deaths and Brazil with 555,383 cases and 31,199 deaths [7].

COVID-19 has an incubation period that varies from 2 days to 14 days and is characterized initially by flu-like symptoms but may further develop to severe coughs, shortness of breath, stomach pain, diarrhea, sore throat, loss of taste and smell, tiredness, muscle aches/pain, repeated shaking with chills, and fever. Severe symptomatology warrants hospitalization in some patients. To date, there is no cure or vaccine for the virus.

Risk factors for COVID-19 include close contact with secretions from an infected person, chronic illness (e.g., asthma, hypertension, kidney disease, lung disease, diabetes), immunosuppression, being male, obesity, older age (60–65 years of age), heart disease, and living or working in nursing homes [3]. Recent statistics suggest disparities in the incidence, prevalence, and mortality rates in COVID-19 among certain populations. 

Many populations disproportionately affected by these risk factors are at increased risk for COVID-19. In the United States, Blacks, Hispanics/Latinx, and Native Americans are disproportionately impacted by COVID-19. For example, Blacks comprise 13% of the U.S. population and, according to the Centers for Disease Control and Prevention (CDC), account for 28% of COVID-19 cases and 33% of hospitalizations, whereas Hispanics/Latinx account for 18% of the U.S. population and 28% of COVID-19 cases [8]. Mortality rates for Blacks and Hispanics/Latinx are also higher [9]. While data for Native Americans are sparse, available data from the Indian Health Service show that in states with higher concentrations of Native Americans, there are disproportionate rates of infection and death [10,11,12]. Similar trends have been observed in other countries [13]. Data from the Office of National Statistics in the UK show that, when controlling for age in England and Wales, Blacks are 4.2 to 4.3 times more likely to die from a COVID-19-related death than Whites. These data also reveal that compared to Whites, Bangladeshis, Pakistanis, Indians, and those of mixed ethnicities are at increased risk of death from COVID-19 [13].

Social determinants underlying health conditions affecting these populations are believed to make them more vulnerable to the virus. These determinants include but are not limited to access to healthcare, economic insecurity, poor neighborhood and housing conditions, and availability of resources. For instance, patterns of social engagement and sense of security and well-being are affected by where people live. Availability of resources that enhance quality of life can also influence health outcomes.

Additionally, stigma in the form of stereotyping and harassment directed toward groups may be associated with the spread of the virus. Pandemics such as COVID-19 create fear and anxiety, which can lead to social stigma toward certain groups, including people who have travelled abroad, people of Asian descent, or even service and healthcare providers. Stigma and discrimination that stems from it can occur when people associate COVID-19 with a nationality, even though not everyone in that nationality is at risk for the disease. Stigma can lead to social avoidance, denial of health care, and perhaps even violence. Understanding how social determinants of health and stigma contribute to the incidence, prevalence, treatment, and mortality associated with COVID-19 may aid in developing more effective interventions to mitigate the transmission of the disease.

## 2. Social Determinants of Health and COVID-19

Social determinants of health refer to “conditions in the places where people live, learn, work, and play that affect a wide range of health risks and outcomes” [14]. These conditions provide the context in which health occurs. COVID-19 is highly transmittable, and mitigation strategies include following healthy hygiene practices, staying at home when sick, practicing physical distancing to lower the risk of disease transmission, and use of a cloth face covering when physical distancing cannot be maintained. When experiencing COVID-19 symptoms, accessibility of testing is also needed to reduce disease spread. Social determinants, such as access to health care, income inequality (e.g., low income wage workers), housing and neighborhood density, and cultural beliefs about testing, may influence COVID-19 incidence and health outcomes in vulnerable populations.

### 2.1. Access to Healthcare

The World Health Organization (WHO) defines primary healthcare as “a whole-of-society approach to health and well-being centered on the needs and preferences of individuals, families, and communities.” [15]. Access to healthcare is a fundamental human right, but the strain that the COVID-19 pandemic places on healthcare systems affects primary care provision for many people.

People who believe they are infected with the COVID-19 virus need to seek testing or immediate medical care. In the U.S., 30 million people do not have health insurance or a primary care provider, and, during the initial COVID-19 period, some testing sites required insurance and a referral from a medical provider [16,17,18]. Blacks and Hispanics/Latinx in the US are less likely to have access to hospitals and pharmacies and wait for days to get urgent care and prescriptions [18]. In some areas, there may be less available testing sites, such as in rural areas compared to larger metropolitan cities [19]. Inadequate access is also driven by a long-standing distrust of the health care system, language barriers, and financial implications associated with missing work to receive care [20].

### 2.2. Housing and Neighborhood Density

The physical structure of communities, such as proximity to resources like grocery stores, green space, the mix of businesses, amenities, and housing, affects the COVID-19 pandemic. High-density housing and group living quarters accelerate transmission of the coronavirus, disproportionately affecting older adults in nursing homes and people with compromised health in overcrowded communities. Blacks, relative to Whites, are more likely to live in neighborhoods with a lack of healthy food options, recreational facilities, safety, and lightning. Blacks are also more likely to live in densely populated areas, further increasing their contact with other people. Blacks represent about a quarter of public transit users, which may affect accessibility to testing sites. These neighborhood characteristics make it more challenging to maintain physical distancing and self-quarantine to curb COVID-19 transmission [21,22].

### 2.3. Income Inequality

According to United Nations data, 55% of the world’s population—four billion people—lack insurance or social assistance, and only about 20% of unemployed people are covered by unemployment benefits [23]. In the US, White workers earn 28 percent more than Black workers and 35 percent more than Hispanic/Latinx workers. Blacks and Hispanics/Latinx are also more likely to have service, transportation, and jobs in sales compared to Whites [24,25]. With COVID-19, members of minority groups may be at greater risk of infection as workers in essential industries who must continue to work despite the outbreaks in their communities or due to their economic situations. Workers without paid sick leave might be more likely to continue to work even when they are ill. This can increase workers’ exposure to other workers who may have COVID-19, or in turn, expose others to them. Gaps in health benefit coverage may also result in workers being forced to go to work when ill. Exacerbating this, job loss during the COVID-19 pandemic is higher for Blacks and Hispanics/Latinx [26]. Results from a recent PEW research survey reveal that Blacks and Hispanics/Latinx are less likely to have savings to cover living expenses for at least 3 months [27]. The related income loss increases the risk of poverty for workers and their families. Such data suggest that these workers and their families may not have access to needed healthcare or necessities, such as food, which may worsen health outcomes.

### 2.4. Cultural Beliefs

Cultural beliefs and perceptions of causes of disease contribute to health behaviors. European nations and the United States have a history of racial bias in medical treatment and research, which in some instances, has led to mistrust of the medical system [28]. This mistrust can be associated with underutilization of health services, limited participation in clinical research studies, and reduction in organ and blood donation [29]. As with many new diseases, with the novel coronavirus, limited information about the disease and mitigation strategies to prevent transmission may intensify cultural beliefs about disease onset and treatment.

For populations at risk for COVID-19, strongly held religious beliefs that God will protect them from disease may result in limited testing and non-compliance with social distancing as a mitigation strategy. Social cohesion and social gatherings are of great importance in many cultures. For example, weekly attendance of a religious service is high among many cultures around the world [30,31]. As a result, measures to impose social and physical distancing may be more challenging. These populations are also more likely to experience negative encounters with healthcare systems which reduces the likelihood they will seek testing for the virus or follow recommended treatment guidelines.

## 3. Stigma and COVID-19

Stigma in the context of health is “the negative association between a person or group of people who share certain characteristics and a specific disease” [32,33]. When a disease is novel and leads to severe symptoms or death, fear, anxiety, and limited knowledge about the disease may lead to stereotyping, discrimination, and labelling toward persons with the disease. Discriminatory behaviors, such as isolation, refusal to provide service, harassment, and bullying, may be experienced by the stigmatized group. Stigma may also affect individuals associated with those with the disease, such as caregivers, family members, those in the same community, or the same racial/ethnic group. Such behaviors may undermine strategies to mitigate the disease and can lead to not getting tested and not practicing healthy behaviors, such as wearing masks to avoid discrimination.

In Africa, during the Ebola pandemic, stigmatization in the form of discrimination, prejudice, and social isolation that arose during the outbreak continued among those who survived [34]. Similarly, in COVID-19 cases in Africa, protective measures, such as wearing a mask, being tested, or the belief of coming into contact with an infected person, have led to people being ostracized, harassed, and isolated from others [35]. This includes healthcare workers, who also experience physical exhaustion and poor mental health outcomes as a function of stigma associated with treating COVID-19 cases [36].

For Asians and Asian Americans, there is an increase in hate crimes in the US due to the coronavirus global outbreak [37]. Since the outbreak of the COVID-19 pandemic, Asians and people of Asian descent have been targets of threats, attacks, bullying and derogatory language in media reports and statements by politicians and on social media platforms where hate speech related to COVID-19 has spread. In the US, a coalition of Asian American groups received almost 1500 reports of incidents of racism, hate speech, discrimination and physical attacks against Asians and Asian Americans, fueling racism and xenophobia against Asians worldwide [38,39].

Prevention strategies, such as wearing a mask, have also led to concerns about racial profiling and harassment of Black American men by law enforcement [40]. Masks or face coverings such as bandanas, may be linked to crime and influence perceptions of criminal activity by Black men [41]. Data from the US Police Shooting Database show that unarmed Black Americans, compared to unarmed White Americans, are three times more likely to be shot by the police [42,43]. Such data underscore concerns by Blacks regarding the use of masks to mitigate COVID-19. Stigmatization may not only impede opportunities to reduce the spread of infection but may inadvertently increase disease transmission and mortality from it.

## 4. Conclusions

In this overview, we address the possible role of social determinants of health and stigma on disparities associated with COVID-19. There are still many unanswered questions. To fully address disparities in COVID-19, greater attention must be paid to collection of data on race and ethnicity. This should also include socioeconomic data, residential status data (i.e., rural vs. urban), institutional racism data, and data on comorbidity at minimum [19,41,44]. As more information is acquired about this disease and antecedents associated with its transmission and mortality rates, strategies for better prevention and intervention may be developed. This is important given that there is currently no vaccine to prevent the onset of COVID-19.

## Figures and Tables

**Table 1:** Global Coronavirus Cases and Deaths ^1^.

Regions/Areas ^2^	Cases	Deaths
Globally	6,194,533	376,320
Africa	108,121	2700
Americas	2,905,432	163,248
Eastern Mediterranean	536,148	12,999
Europe	2,175,941	182,416
South-East Asia	283,845	8000
Western Pacific	184,305	7044

^1^ Source: World Health Organization, COVID-19 Situation Report. **^2^** Americas: the continents of North and South America; Eastern Mediterranean: east of the Mediterranean Sea in Western Asia; South-East Asia: regions between the Indian Ocean and Pacific Sea; Western Pacific: countries in the Pacific, Oceania, and parts of Asia.
